# Dermoscopic Patterns in Granulomatous Diseases: A Narrative Review

**DOI:** 10.3390/jcm15207821

**Published:** 2026-10-10

**Authors:** Sanjana Sara Sunil, Katarzyna Korecka, Adriana Polańska

**Affiliations:** 1 Center for Medical Education in English, Poznań University of Medical Sciences, 60-512 Poznań, Poland; 2 Department of Dermatology and Venereology, Poznań University of Medical Sciences, 60-355 Poznań, Poland

**Keywords:** dermoscopy, granulomatous disorders, non-invasive imaging

## Abstract

Dermoscopy has gained increasing recognition in recent years as a valuable non-invasive supplementary tool in diagnosing various skin disorders. However, a comprehensive overview of the distinguishing features of various cutaneous granulomatous diseases remains limited. This narrative aims to summarize the overlapping dermoscopic patterns reported across various diseases such as leprosy, cutaneous tuberculosis, *Mycobacterium marinum*, granuloma annulare, necrobiosis lipoidica, sarcoidosis, granulomatous variant of rosacea, annular elastolytic giant cell granuloma, and lupus miliaris disseminatus faciei. Across these conditions, several overlapping dermoscopic features were observed including, yellow-orange structureless areas, white structureless areas, linear, dotted and branching vascular patterns. These shared patterns can limit early recognition and diagnostic specificity. When interpreted alongside clinical features, dermoscopy may help narrow the differential diagnosis. However, dermoscopy alone is inadequate for definitive diagnosis; histopathology remains the gold standard. This narrative highlights the overlapping dermoscopic features of various cutaneous granulomatous diseases, emphasizing its role as a complementary tool.

## 1. Introduction

Dermoscopy is a useful, noninvasive technique applied in enabling rapid diagnosis for many skin disorders [1]. In recent years, it has attained its increasing recognition as a complementary tool for diagnosing various cutaneous granulomatous disorders (CGDs) [2]. However, the current understanding of dermoscopic features of CGDs is predominantly based on small case reports and limited observational studies, highlighting the need for large-scale prospective data.

CGDs, defined by granuloma formation in the skin, are typically classified into infectious and noninfectious forms [3]. The infectious forms include Leprosy and *Mycobacterium marinum* infection [4,5]. The noninfectious forms of CGDs such as cutaneous sarcoidosis, granuloma annulare, necrobiosis lipoidica, and granulomatous rosacea often show overlapping dermoscopic features [6]. Dermoscopy serves as a noninvasive complementary tool in the evaluation of skin condition and as a bridge between clinical assessment and histopathological examination. By identifying characteristic dermoscopic features—including scaling, color variation, follicular criteria, and vascular patterns, clinicians can better characterize a range of granulomatous inflammatory skin conditions when these findings are interpreted alongside the clinical presentation and histopathology [7].

Since CGDs frequently share overlapping dermoscopic features, accurate and timely diagnosis is essential. Early detection allows clinicians to guide appropriate management and prevent disease progression. While histopathology remains the gold standard, dermoscopy offers a valuable noninvasive complement, enabling clinicians to identify supportive dermoscopic features and select the most suitable biopsy site.

The aim of this narrative review is to summarize the current knowledge on the use of dermoscopy in CGDs.

## 2. Methods

This narrative review was conducted to summarize the published dermoscopic features of infectious and noninfectious granulomatous skin diseases. A literature search was performed using PubMed and included English-language publications relevant to dermoscopy from 2010 to 2026. The search strategy combined “dermoscopy” or “dermatoscopy” with disease-specific terms for the granulomatous skin disorders covered in this review, using the Boolean operator AND. For example, “leprosy AND dermoscopy” was used, and similar combinations were applied to the other diseases discussed in the review. Studies were considered eligible if they reported dermoscopic findings in the granulomatous skin diseases included in this review. Observational studies, case series, and case reports were considered. Additional older publications were included when necessary to provide relevant clinical and general background information on the diseases. The search and selection process was performed by reviewing the identified publications for relevance to the objectives of the review.

## 3. Dermatoscopic Features by Entity

### 3.1. A. Infectious Granulomatous Diseases

#### 3.1.1. Leprosy

Leprosy, also known as Hansen disease, is a longstanding granulomatous infection caused by *Mycobacterium leprae* (*M. leprae*) or *Mycobacterium lepromatosis*, with a wide range of clinical manifestations [8]. It can be classified using the Ridley–Jopling system which categorizes patients according to their clinical presentation and host immune response [9]. The classification ranges from tuberculoid (TT) to lepromatous leprosy (LL), with borderline forms in between. Tuberculoid leprosy typically presents with a few well-demarcated hypopigmented erythematous plaques, whereas lepromatous leprosy presents with multiple diffuse skin infiltrates of papulonodular lesions [9,10,11]. The intermediate forms include borderline tuberculoid (BT), mid-borderline (BB), and borderline lepromatous (BL) that may exhibit annular plaques with poorly demarcated borders [10]. Dermoscopy of tuberculoid leprosy reveals hair and pigment depletion, along with loss of white dots corresponding to sweat glands. In contrast, lepromatous leprosy reported dermoscopic features including white, scaly skin accompanied by reduced hair density and hypopigmentation [10]. Type 1 leprosy reaction, also known as a reversal reaction, is characterized by a type IV hypersensitivity response directed against *M. leprae* antigens [12]. It most commonly occurs in the borderline tuberculoid form of leprosy [10]. The presence of widespread redness was suggestive of a type 1 leprosy reaction, whereas the absence of prominent vascular changes may provide a supportive clue against a lepra reaction [7]. Ankad et al. [11] evaluated dermoscopic patterns in 12 patients with borderline tuberculoid leprosy. White areas were present in all 12 patients (100%), yellow globules were observed in eight (66.7%). Decreased white dots, corresponding to sweat duct openings, were observed in six patients (50%), and branching vessels in four patients (33.3%) [11]. Mohta et al. [4] similarly reported white structureless areas across the leprosy spectrum, with additional dermoscopic features described in type 1 reactions, including violaceous structureless areas, large telangiectatic vessels, follicular plugging, star-shaped silvery-white scaling, and white globules [4]. In addition to cutaneous dermoscopic findings, a study conducted by Gunawan et al. [13] highlighted the nail changes observed in BT, BL, BB and LL patients using a 10× dermoscope. The characteristic nail changes described in the aforementioned study were: longitudinal ridging, along with transverse lines, onycholysis, leukonychia, onychorrhexis, subungual hemorrhage and hyperkeratosis [13]. Moreover, a prospective observational study by Sahu et al. [1] involving 70 clinically suspected leprosy patients reported a dermoscopic-histopathological correlation of more than 87% across most leprosy subtypes, whereas the correlation was only 25% in mid-borderline leprosy. These findings suggest that dermoscopic features may show substantial concordance with histopathological findings in some forms of leprosy, although their correlation may vary according to the clinical subtype.

#### 3.1.2. Cutaneous Tuberculosis (*Mycobacterium tuberculosis*)

Cutaneous tuberculosis (CT) is an uncommon manifestation of extrapulmonary tuberculosis [14]. CT includes various forms such as lupus vulgaris, scrofuloderma, tuberculosis verrucosa cutis, lichen scrofuloderma and papulonecrotic tuberculoid [15]. In a case series by Jindal et al. [15], lupus vulgaris was the most common variant of CT, typically observed in the head and neck region. It typically presents as brownish-red macules or papules forming larger plaques [16]. Dermoscopy revealed yellow-orange structureless areas (100%), linear/dotted vessels (100%) and white areas (84.6%) in 13 patients [15]. Similarly, in a study by Ankad et al. [17], 19 patients with lupus vulgaris showed similar white structureless areas (100%), white scales (100%), and telangiectasias in 16 patients (84.21%). Scrofuloderma, also known as tuberculosis cutis colliquativa, clinically observed as cold abscesses or subcutaneous nodules in association with an underlying tuberculous lymph node [18], was seen in five patients in a study by Jindal et al. [15], all of whom consistently exhibited white structureless areas and linear vessels. Less common variants of CT were also reported. Tuberculosis verrucosa cutis under dermoscopy, reported in two patients, demonstrated a papillated surface with dirty white scales, lichen scrofuloderma revealed white globules surrounded by erythema in one patient and papulonecrotic tuberculoid seen in one patient, exhibited keratin plugs and yellow-orange structureless areas [15].

#### 3.1.3. *Mycobacterium marinum*

*Mycobacterium marinum* is a nontuberculous opportunistic bacterium affecting the skin by inducing granulomatous lesions [19]. It typically occurs in areas of previously injured skin that have been exposed to contaminated water sources, including through occupational contact with non-chlorinated water [19]. Yao et al. [5], in a study of 27 lesions, and Conforti et al. [19], in a case report, reported similar findings: lesions predominantly affecting the hands and forearms and showing a characteristic dermoscopic pattern of yellow-orange structureless areas, which was present in 85.2% of lesions in the larger study [5]. Histologically, these orange areas corresponded to granulomatous inflammation within the dermis [19]. Both studies also identified a range of vascular patterns, including long hairpin vessels (81.5%), dotted vessels, and looped concentric monomorphic vessels, with corkscrew and comma-shaped vessels noted as less common findings [5,19]. The findings from these studies suggest that the orange structureless areas along with various vascular patterns may provide supportive dermoscopic clues in *M. marinum* infection. An overview of clinical and dermoscopic key features of infectious granulomatous diseases discussed in this review is provided in Table 1.

### 3.2. B. Noninfectious Granulomatous Diseases

#### 3.2.1. Granuloma Annulare

Granuloma annulare (GA) is a cutaneous disorder predominantly affecting the feet, dorsal hands, or both. Clinically, it presents as skin-colored annular plaques with a raised, rope-like border and central clearing [20,21]. The localized form is often asymptomatic. GA can also present as a generalized variant which includes disseminated papular and atypical generalized subtypes that may require medical evaluation to identify potential underlying causes [21]. Other variants are subcutaneous and perforating forms [21]. Subcutaneous GA manifests as non-inflamed nodules, most commonly on the lower legs, hands, head, and buttocks, and primarily affects children and young adults. The perforating GA typically presents as umbilicated papules or pustules [22].

**Figure 1:**
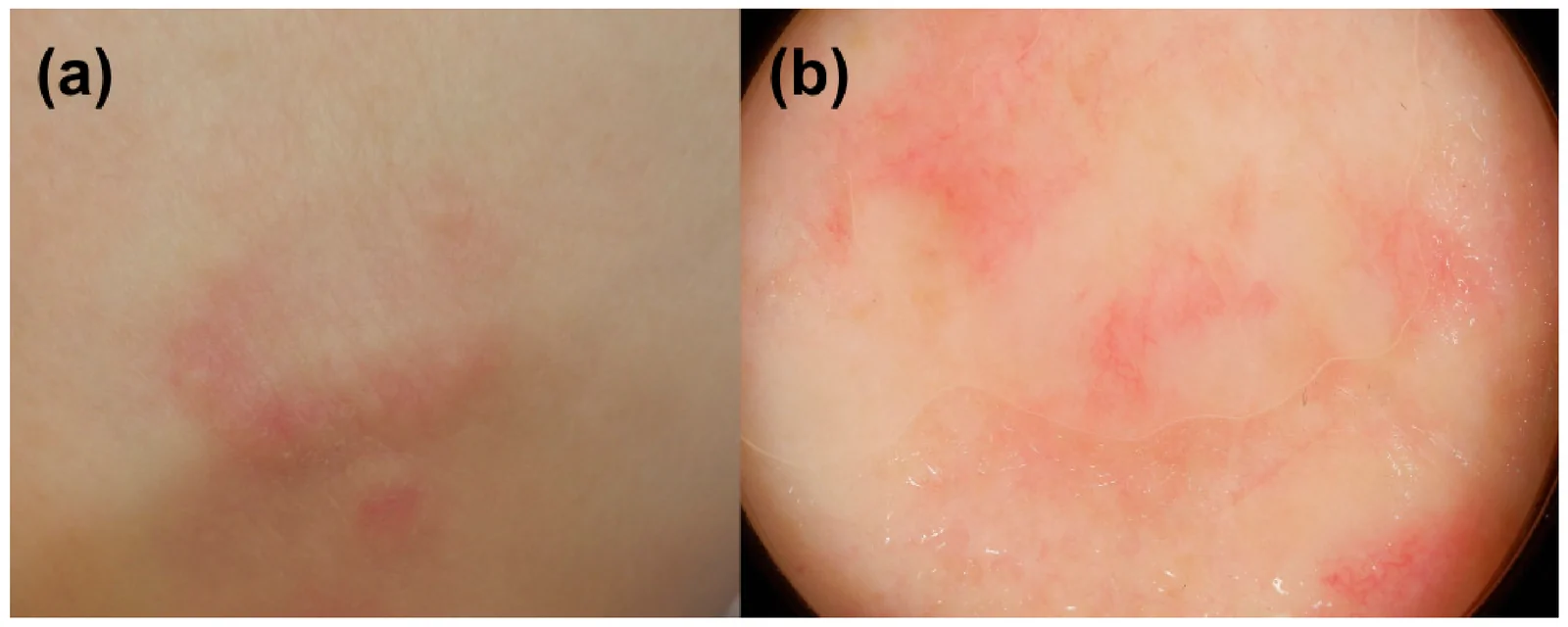
(**a**) The clinical image of granuloma annulare. (**b**) Dermoscopic features of granuloma annulare, revealing yellowish-orange structureless areas along with branching vessels.

The dermoscopic appearance of GA is generally independent of clinical presentation but differs according to histologic subtype [21]. Pellicano et al. [23] evaluated 12 patients with granuloma annulare lesions. On dermoscopy characteristic peripheral, homogeneous orange-reddish borders were seen [23]. In these 12 patients the most common feature was the presence of white areas, mainly on the arms and less commonly on the trunk and back [23]. Similarly, Errichetti et al. [21] in a correlational study of 25 lesions, reported that histopathological examination revealed interstitial granuloma in 11 cases and palisading granulomas in 14 cases, with dermoscopic yellowish-orange areas showing a strong correlation with the palisading granuloma subtype. Common dermoscopic features included dotted, linear or branching vessels against a pinkish-red background along with whitish and/or yellowish-orange areas [21]. However, the presence of structureless yellowish-orange areas on dermoscopy was significantly correlated to palisading granuloma, suggesting a possible association between this dermoscopic feature and the palisading pattern [21]. The clinical image and dermoscopic features of granuloma annulare are illustrated in Figure 1.

#### 3.2.2. Necrobiosis Lipoidica

**Figure 2:**
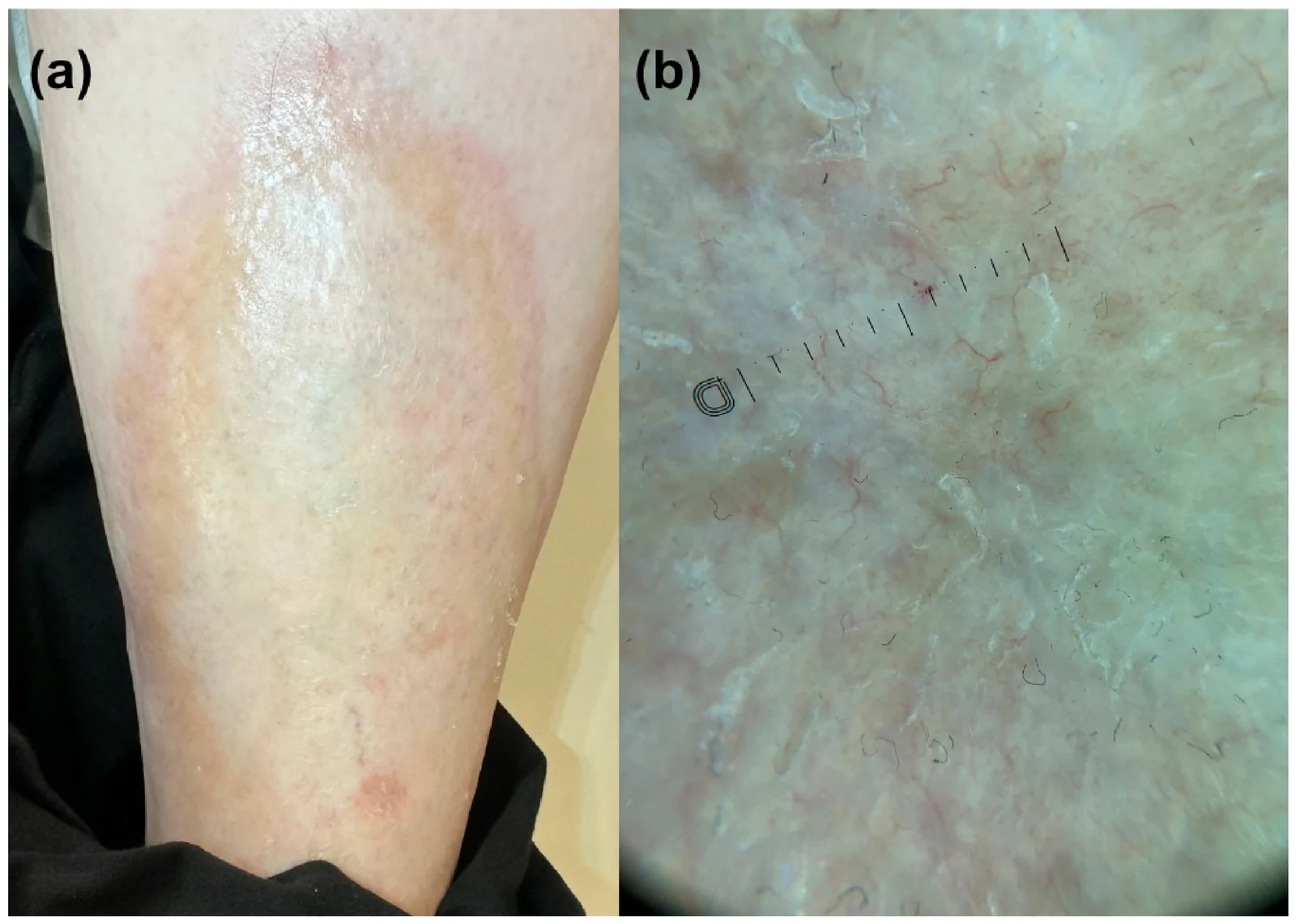
(**a**) Clinical image demonstrating yellow-orange areas of necrobiosis lipoidica. (**b**) Dermoscopic features of necrobiosis lipoidica showing white structureless areas with serpentine telangiectasias.

Necrobiosis lipoidica (NL) is a rare cutaneous disorder that presents with or without ulcerations and is predominantly observed in females and individuals with diabetes mellitus [23]. It is clinically characterized by the presence of erythematous papules or plaques that extend centrifugally and eventually develop into a brownish-yellow plaque with central atrophy, typically seen in the pretibial region [24,25]. A retrospective study conducted by Pellicano et al. [23] included 12 patients with NL, predominantly women, with a mean age of 48.5 years. Most patients presented with the disseminated variant, while only two had solitary lesions. Dermoscopic features included well-focused, serpentine telangiectasias typically observed over a structureless whitish background, with yellow-orange areas noted in few cases [23]. In a case of disseminated perforating NL, Saber et al. [26] reported a yellowish-pinkish structureless background, well-focused linear vessels, comedo-like openings, white structureless areas, follicular plugging, and silvery-white lamellated scales. Similarly, in a case report by Shrestha et al. [27], dermoscopic examination revealed linear vessels with branching against a yellow structureless area, along with white streaks. Figure 2 presents the clinical and dermoscopic findings observed in a patient with necrobiosis lipoidica.

#### 3.2.3. Sarcoidosis

**Figure 3:**
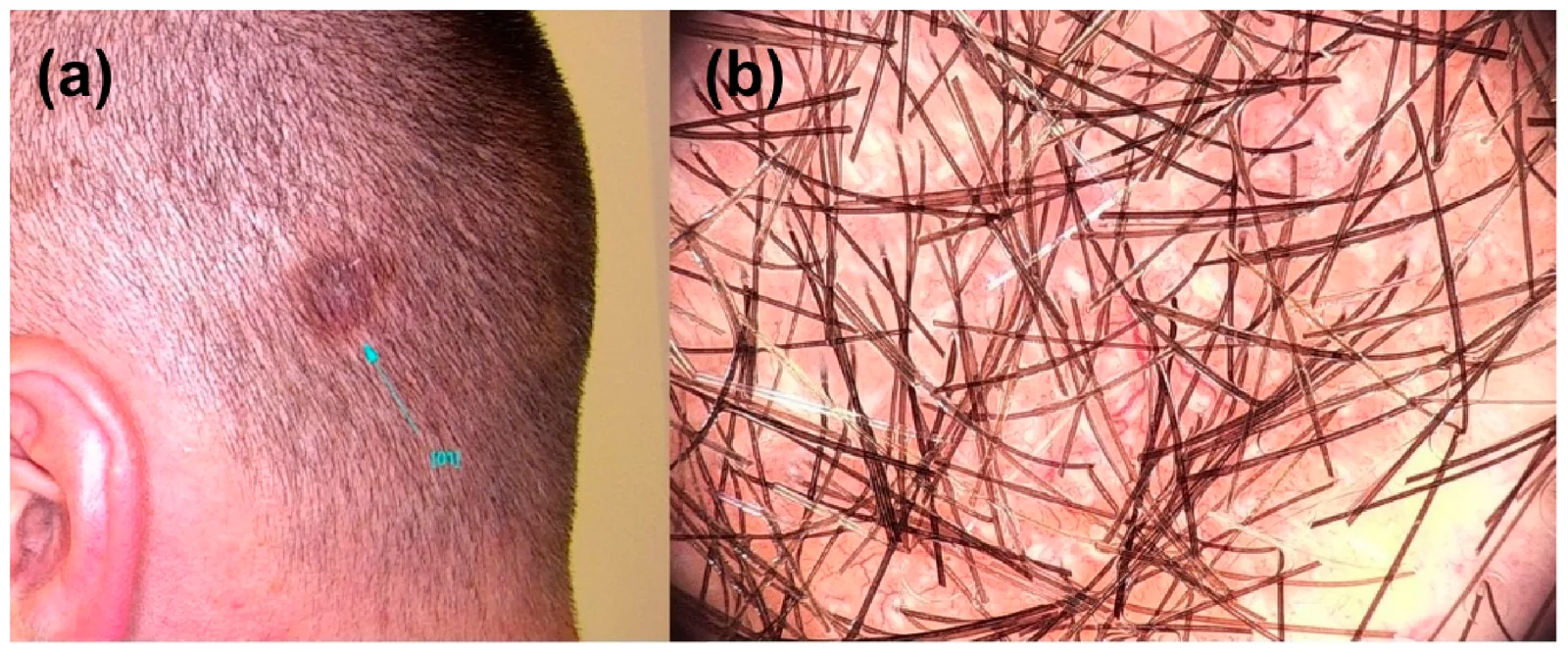
Clinical and dermoscopic features of cutaneous sarcoidosis on scalp. (**a**) Clinical image of the lesion on the scalp. (**b**) Dermoscopy of lesion revealing translucent orange macule with linear vessels and shiny white lines.

Sarcoidosis is a granulomatous disorder of uncertain origin whose cutaneous form is well known for its ability to mimic a broad range of skin diseases [28]. Cutaneous sarcoidosis (CS) presents across several morphological subtypes, including maculopapular, nodular, annular, scar, lupus pernio, and plaque variants [29]. Of these, lupus pernio is the most well-characterized, occurring in 50% of patients with pulmonary sarcoidosis and manifesting as slowly developing, indurated, violaceous lesions on the nose, lips, cheeks, and ears, often accompanied by swelling of the fingers and toes [30,31]. Cutaneous involvement is seen in approximately one-third of patients, and yellow-orange areas or globules are recognized as the hallmark dermoscopic feature of granulomatous infiltration in CS [28]. Dermoscopic findings vary by lesion type: papular lesions typically show yellow-orange areas, plaque and psoriasiform lesions display pale orange coloration, and pigmented purpuric-like lesions exhibit yellow areas with an accentuated reticular pattern [28]. In a retrospective study by Liu et al. [32], several lesions were initially misdiagnosed clinically as lupus vulgaris and discoid lupus erythematosus; however, dermoscopy identified characteristic translucent orange ovoid structures, linear vascular structures, and central scarring with bright white streaks, with the diagnosis later confirmed histopathologically as cutaneous sarcoidosis. Notably, in a case report by Boda et al. [33], a patient presenting with a shiny, indurated scalp plaque without pruritus or bleeding showed diffuse yellow-orange and pink structureless areas with monomorphic linear vascular structures on dermoscopy. These observations highlight the supportive role of dermoscopy in the early recognition of cutaneous sarcoidosis, particularly when clinical presentation is non-specific, while histopathology remains the diagnostic gold standard. The clinical and dermoscopic features of cutaneous sarcoidosis are illustrated in Figure 3.

#### 3.2.4. Granulomatous Variant of Rosacea

**Figure 4:**
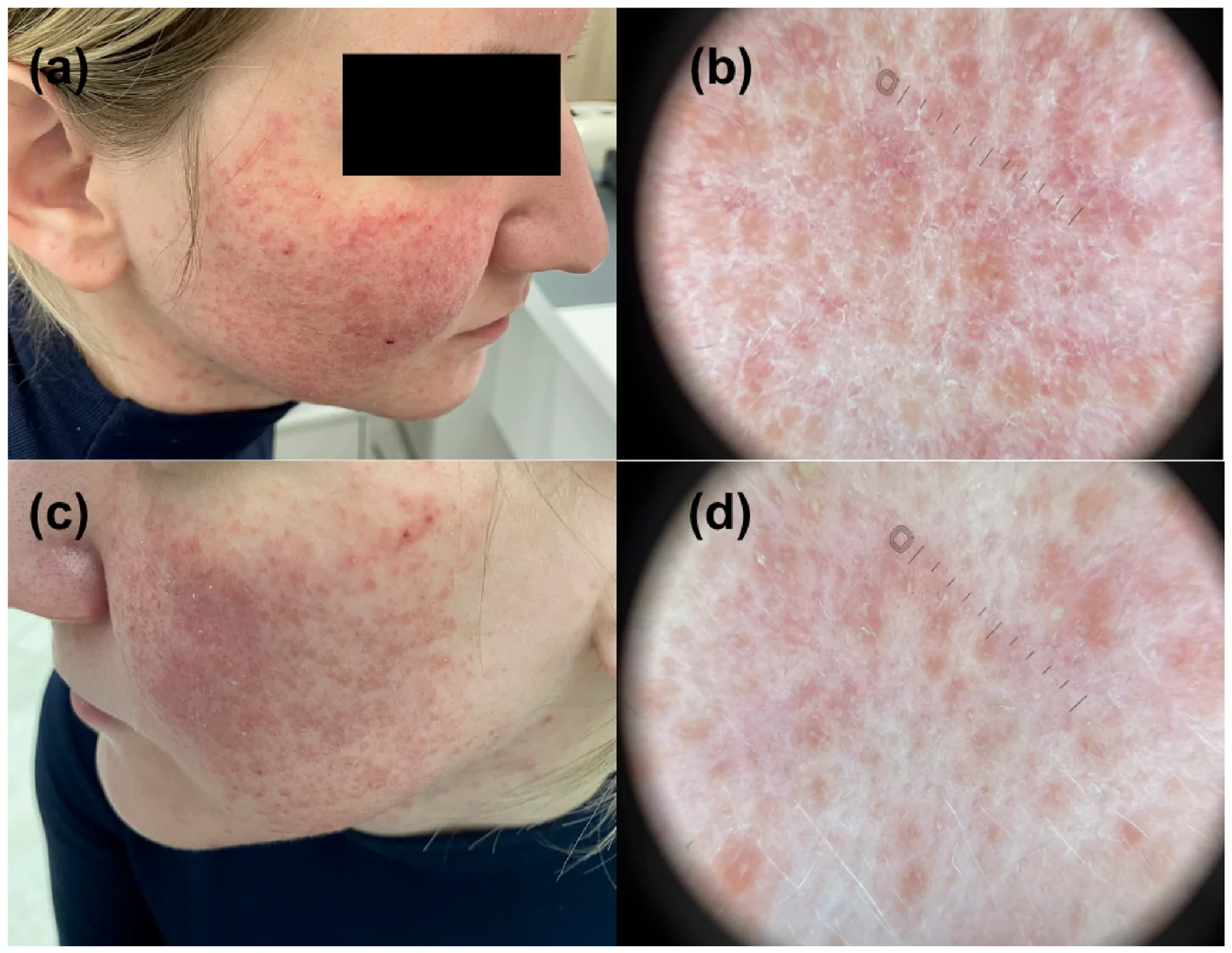
(**a**,**c**) Clinical images of granulomatous rosacea. (**b**,**d**) Dermoscopy revealed focal orange structureless areas along with whitish areas.

Rosacea is a chronic inflammatory skin disorder affecting the face, classified into the classical clinical subtypes: erythematotelangiectatic, papulopustular, phymatous, ocular, and granulomatous [34,35]. While dermoscopic features such as polygonal vascular networks and follicular plugs are characteristic of classical rosacea [35,36,37], a study by Stefanou et al. [38] involving 85 patients identified considerable overlap in dermoscopic findings between classical and granulomatous subtypes. Shared features included linear and linear-branched vessels, erythema, reticular vascular patterns (both regular and patchy), whitish areas, and follicular changes such as plugs, perifollicular orange coloration, and pustules. Notably, focal orange structureless areas were predominantly observed in granulomatous rosacea, which may help distinguish it from the classical form. This overlap was further illustrated by Kelati et al. [39] in a case report of a 54-year-old patient with granulomatous rosacea exhibiting features typical of classical rosacea, including a polygonal vascular network, whitish areas, and white-grayish plugs surrounded by an erythematous halo; histological examination confirmed granulomatous inflammation and the presence of *Demodex* spp. The clinical and dermoscopic features of granulomatous rosacea are illustrated in Figure 4.

Demodex mites are implicated in the pathogenesis of both rosacea and demodicosis. Although standardized skin surface biopsy (SSSB) has historically been the gold standard for mite detection, it is invasive and time-consuming. Ünal et al. [40] demonstrated that UV dermoscopy offers a viable non-invasive alternative, revealing fluorescent bright blue dots corresponding to Demodex folliculorum, with counts exceeding 11 dots reported as a potential diagnostic threshold in that study. In a cross-sectional study of 206 patients, Khachani et al. [41] showed that dermoscopy detected features not apparent on clinical examination alone, particularly in patients with darker phototypes, in whom erythema and pustules were frequently concealed. Pigmented areas were significantly more prevalent in dark phototypes (*p* = 0.003), whereas orange structureless areas (*p* = 0.027) and crusts (*p* = 0.035) were more common in light phototypes. These findings collectively support the use of dermoscopy as a non-invasive complementary tool to identify subtle lesions warranting biopsy and to complement histopathological assessment in the clinical diagnosis of rosacea.

#### 3.2.5. Annular Elastolytic Giant Cell Granuloma

**Figure 5:**
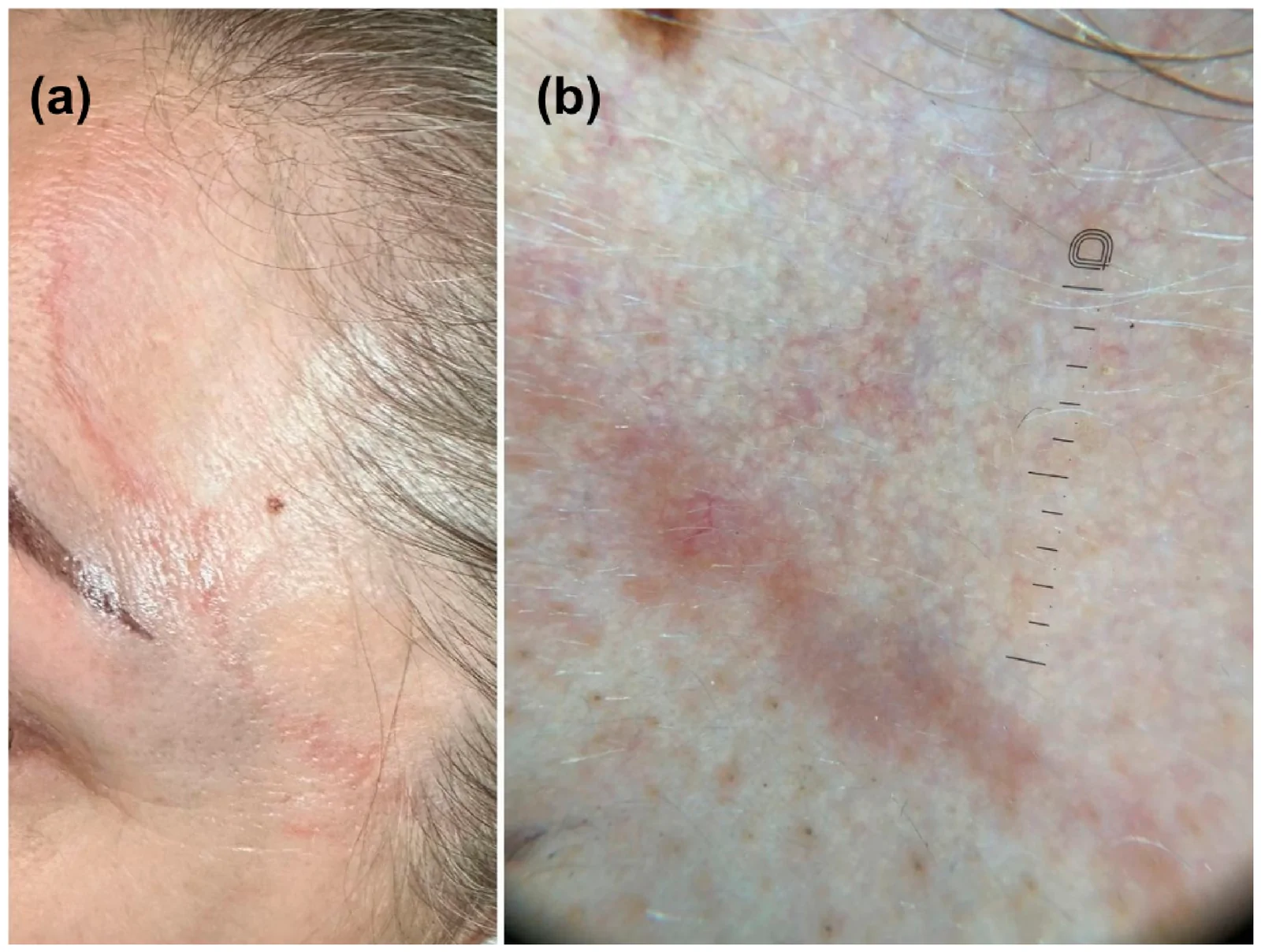
(**a**) Clinical image of Annular Elastolytic Giant Cell Granuloma. (**b**) Dermoscopy revealed linear branching vessels with whitish structureless background.

Annular Elastolytic Giant Cell Granuloma (AEGCG) is a rare cutaneous granulomatous disease that predominantly affects the sun-exposed regions [42]. A case report by Gomes et al. [42] demonstrated a patient with alopecic pink patches along with an erythematous border on physical examination. Dermoscopy revealed empty follicular ostia against a diffuse erythemo-whitish background, fine linear branching vessels and multiple yellow-orange follicular dots. Applying increased dermoscopic pressure produced a whiter background that enhanced the visibility of orange dots [42]. Histopathology performed thereafter confirmed mid-dermal granulomatous infiltrates with multinucleated giant cells containing elastic fibers, confirming the diagnosis of AEGCG [42]. In another case reported by Chauhan et al. [43], a patient presenting with raised lesions on the chest, dermoscopy showed structureless yellow-brown and white structures, along with multiple linear irregular and reticular vessels against a pale pink background, corresponding to dermal granulomatous infiltrates on histopathology [43]. Both cases reported whitish areas with linear branching vascular structures. Additional findings included pressure-dependent follicular changes, orange-yellow dots and lesions with yellow-brown areas. Therefore, these observations suggest that dermoscopy as a tool may complement histopathology in the clinical diagnosis of AEGCG. The clinical and dermoscopic features of AEGCG are illustrated in Figure 5.

#### 3.2.6. Lupus Miliaris Disseminatus Faciei

Lupus miliaris disseminatus faciei (LMDF) is an uncommon disorder occurring mainly on the face and eyelids, identified by yellowish-red papules, and often extending to the neck [44,45]. A small case series of three patients highlighting their dermoscopic features was reported by Litaiem et al. [46]. Dermoscopy in the first patient revealed sparse follicular keratotic plugs (FKP) accompanied by linear telangiectatic vessels and white scales on an erythematous and yellow-orange background. In the second patient, FKP were surrounded by an orange perifollicular halo with dotted vessels. In the third patient, FKP were surrounded by white structures, with numerous yellow dots and comma-shaped vessels also identified. The yellow dermoscopic background was histopathologically correlated with early cases of dermal granulomatous formation [46]. Similarly, Dudani et al. [47] reported structureless yellow-red areas, follicular plugs, and telangiectasias on dermoscopy in a patient with LMDF. Dhanta et al. [48] likewise identified follicular plugs and white streaks against an orange-brown and erythematous background.

#### 3.2.7. Childhood Granulomatous Periorificial Dermatitis

Childhood granulomatous periorificial dermatitis (CGPD) is a self-limiting condition of unknown etiology, typically occurring in children up to 18 years of age. It clinically manifests around the mouth, nose and eyes as small, monomorphic papular ranging in color from flesh-colored to yellow-brown papules, majority of cases being asymptomatic [49,50]. In a case report of a 16-year-old Caucasian boy by Lacarrubba et al. [51], polarized dermoscopy revealed several roundish, homogenous orange areas against a red background along with scaling. Reflectance confocal microscopy in the same case exhibited a thinned epidermis, absence of dermal papillae and multiple bright structures in upper dermis around the hair follicles of varying sizes [51]. In another case report by Hayakawa et al. [52], an 11-year-old boy presenting with erythematous papules was evaluated with dermoscopy, which revealed orange areas, linear blood vessels, and scaling against a reddish background. Following discontinuation of all topical treatments, perifollicular white dots became more prominent [52]. Notably, this case demonstrated that the orange areas with white dots on dermoscopy corresponded to the perifollicular noncaseating granulomas later observed on histopathology, supporting an association between these dermoscopic findings and perifollicular noncaseating granulomas [52]. Although CGPD usually occurs in children, Goyal et al. [53] reported a case of a 40-year-old female with multiple raised lesions around the mouth which was later diagnosed as CGPD. Dermoscopy exhibited similar findings as the above cases, such as numerous vessels including linear, linear-curved and with branches, erythematous to orange structureless areas along with white dots and scaling with histopathologically confirming non-caseating granulomas [53]. A summary of key dermoscopic features of non-infectious granulomatous diseases discussed in this review is presented in Table 2.

## 4. Discussion

The available dermoscopic evidence across granulomatous skin diseases is limited by small sample sizes and predominantly observational study designs, with much of the literature consisting of case reports and small case series. These limitations restrict the ability to determine the diagnostic performance of individual dermoscopic findings. Although several studies have reported associations between dermoscopic and histopathological findings, these correlations should not be interpreted as evidence of diagnostic specificity or accuracy. Overlapping dermoscopic features, particularly yellowish-orange areas, may occur in different granulomatous disorders, limiting their diagnostic value. Therefore, dermoscopy should be considered a complementary tool that may provide additional clues and help narrow the clinical differential diagnosis rather than a substitute for histopathological, microbiological, or other appropriate investigations. Further prospective, multicenter studies with larger patient populations are needed to establish the diagnostic performance and reproducibility of individual dermoscopic findings.

## 5. Conclusions

Dermoscopy is a useful complementary tool in the evaluation of granulomatous skin disorders, particularly when clinical features overlap and the differential diagnosis is broad. Although certain dermoscopic patterns may provide clues to the underlying diagnosis and help narrow the clinical differential diagnosis, considerable overlap exists among different granulomatous conditions. The yellowish-orange areas frequently described in granulomatous disorders are not entirely specific and may also be observed in other inflammatory conditions. Therefore, dermoscopic findings should always be interpreted in conjunction with the clinical presentation and, when indicated, confirmed by histopathological, microbiological, and other appropriate investigations. Dermoscopy may also assist in identifying suitable lesions or areas for biopsy but should not be considered a substitute for definitive diagnosis.

## Figures and Tables

**Table 1:** Infectious granulomatous diseases and their dermoscopic features.

Infectious Granulomatous Disease	Clinical Presentation/Subtype	Dermoscopic Key Features
Leprosy	Tuberculoid type	Yellowish-orange areas, diminished pigment network, vascular structures [1]
Leprosy	Lepromatous type	White scaling, partial loss of hair follicles [7]
Leprosy	Borderline Tuberculoid	White areas, partial loss/decreased hair density, yellow globules, decreased or absent white dots (sweat ducts opening) and branched vessels (variably reported) [7,11]
Leprosy	Type 1 reaction	Diffuse erythematous background, branched telangiectatic vessels, sparse hair follicles, scaling [7]
Leprosy	Nail involvement	Longitudinal ridging, transverse lines, leukonychia, onycholysis, hyperkeratosis, subungual hemorrhage [13]
*Mycobacterium tuberculosis*	Lupus vulgaris	Yellow-orange structureless areas, linear/dotted vessels and white structureless areas [15]
*Mycobacterium tuberculosis*	Scrofuloderma	White structureless areas and linear vessels [15]
*Mycobacterium tuberculosis*	Tuberculosis verrucosa cutis	Papillated surface, dirty white scales [15]
*Mycobacterium tuberculosis*	Lichen scrofuloderma	White globules surrounded by erythema [15]
*Mycobacterium tuberculosis*	Papulonecrotic tuberculoid	Yellow-orange structureless areas and keratin plugs [15]
*Mycobacterium marinum*	*Mycobacterium marinum* infection	Orange/yellow structureless areas, long hairpin vessels, dotted vessels, and looped concentric monomorphic vessels [5,19]

**Table 2:** Non-infectious granulomatous diseases and their dermoscopic features.

Non-Infectious Granulomatous Disease	Dermoscopic Background Color	Dermoscopic Key Features
Granuloma Annulare [21,23]	homogeneous orange-red	Dotted, linear, or branching vessels
Necrobiosis lipoidica [23,26,27]	homogeneous whitish background	Serpentine telangiectasias
Cutaneous sarcoidosis [28,32,33]	1. Papular lesion—yellow orange areas 2. Plaque/psoriasiform lesions—pale orange 3. Pigmented purpuric dermatosis-like lesion—yellow areas with accentuated reticular pattern	Linear vascular structures, central scarring, and white streaks
Rosacea 1. Classical Rosacea [35,36,37] 2. Granulomatous Rosacea [38,39]	1. Yellow, red halos 2. Erythematous/red halo	1. Vascular polygons, follicular plugs 2. Polygonal network of vessels, whitish areas, white grayish plugs
Annular Elastolytic Giant Cell Granuloma [42,43]	Yellow-brown structureless areas, pale pink background	Irregular and reticular vessels, white areas
Lupus miliaris disseminatus faciei [46,47,48]	Yellow-orange or orange-brown background	Comma-shaped vessels, follicular plugs
Childhood granulomatous periorificial dermatitis [51,52,53]	Red or erythematous background	Orange structureless areas, linear vessels (including curved and branching forms), scaling

## Data Availability

No new data were created or analyzed in this study. Data sharing is not applicable to this article.
